# Controllable Acoustic Mixing of Fluids in Microchannels for the Fabrication of Therapeutic Nanoparticles

**DOI:** 10.3390/mi7090150

**Published:** 2016-09-02

**Authors:** Christoph Westerhausen, Lukas G. Schnitzler, Dominik Wendel, Rafał Krzysztoń, Ulrich Lächelt, Ernst Wagner, Joachim O. Rädler, Achim Wixforth

**Affiliations:** 1 Chair of Experimental Physics I, University of Augsburg, Universitätsstraße 1, 86519 Augsburg, Germany; lukas.schnitzler@physik.uni-augsburg.de (L.G.S.); achim.wixforth@physik.uni-augsburg.de (A.W.); 2 Nanosystems Initiative Munich, Schellingstraße 4, 80799 Munich, Germany; dominik.wendel@cup.uni-muenchen.de (D.W.); r.krzyszton@physik.uni-muenchen.de (R.K.); ulrich.laechelt@cup.uni-muenchen.de (U.L.); ernst.wagner@cup.uni-muenchen.de (E.W.); raedler@lmu.de (J.O.R.); 3 Augsburg Center for Innovative Technologies (ACIT), University of Augsburg, Universitätsstraße 2, 86159 Augsburg, Germany; 4 Pharmaceutical Biotechnology, Department of Pharmacy, Ludwig-Maximilians-Universität, 81377 Munich, Germany; 5 Faculty of Physics, Ludwig-Maximilians-Universität, Geschwister-Scholl-Platz 1, 80539 Munich, Germany

**Keywords:** microfluidic mixing, lab-on-a-chip, surface acoustic waves, acoustic streaming, polyplexes, lipoplexes, siRNA, plasmid DNA (pDNA), polyethylenimine (PEI), mono-nucleic acid/lipid particles (mNALPs), therapeutic nanoparticles

## Abstract

Fifteen years ago, surface acoustic waves (SAW) were found to be able to drive fluids and numerous applications in microfluidics have been developed since. Here, we review the progress made and report on new approaches in setting-up microfluidic, continuous flow acoustic mixing. In a microchannel, chaotic advection is achieved by generation of a SAW driven fluid jet perpendicular to the mean flow direction. Using a high speed video camera and particle image velocimetry, we measure the flow velocities and show that mixing is achieved in a particularly controllable and fast way. The mixing quality is determined as a function of system parameters: SAW power, volume flux and fluid viscosity. Exploring the parameter space of mixing provides a practical guide for acoustic mixing in microchannels and allows for adopting conditions to different solvents, as e.g., required for the generation of nanoscale particles from alcoholic phases. We exemplarily demonstrate the potential of SAW based continuous flow mixing for the production of therapeutic nucleic acid nanoparticles assembled from polymer and lipid solutions.

## 1. Introduction

Mixing microliter volumes of liquids is a challenging task, as inertia effects are negligibly small compared to friction. At low Reynolds numbers, as they occur in most microfluidic applications, mixing is diffusion-limited and hence very slow. To overcome this limitation, several different types of micromixers were presented previously. In general, micromixers can be classified into two groups, passive and active ones [1]. For passive micromixers, there exist rather simple geometries like Y- or T-shapes [2] but also more complex ones, e.g., the herringbone mixer [1]. All passive methods have the problem of long mixing times and channel lengths in common. In contrast, we here present an active micromixer using surface acoustic waves (SAW).

In our group, acoustic streaming and its application for mixing and application to biophysical questions has a long standing tradition, starting with the demonstration of the ability of SAW to actuate and process the smallest amounts of fluids on the planar surface of a piezoelectric chip in 2003 [3,4]. There, we already demonstrated the possibility for lithographical surface modifications to create virtual walls and vessels and presented a variety of assays on a chip that have already been realized. The flow profile in a capillary gap and the pumping efficiency of an acoustic micropump employing propagative SAW and Rayleigh streaming [5,6,7] was investigated both experimentally and theoretically [8]. The streaming velocity was found to depend on the applied linear power and to decrease quickly with the distance from the chip surface. Later in a short letter [9], we demonstrated the application of fast acoustic mixing at low Reynold’s numbers by combining simple Y-shaped channels with a SAW-chip. This early principle was taken up by other groups [10] and is the basis of the in-depth studies presented in the present manuscript. Since then, the number of applications of SAW microfluidics has continuously increased [11,12,13,14].

Our work on acoustic mixing was complemented by cooperative, theoretical and modeling investigations with the Hänggi group demonstrating a striking similarity to the experimental results with model calculations of the flow patterns and the advective transport by applying a raytracing algorithm. The presented concept can be transferred to acoustic streaming systems with different sound sources, like bulk acoustic waves [15]. We furthermore demonstrated the feasibility of this technique in two different modes of operation. For both modes, optimal frequencies characterizing the relevant stretching-folding duty cycles causing the chaotic advection were identified in experimentally accessible frequency regimes [16]. That work presents an approach for determining the streaming patterns that are generated by Rayleigh SAW in arbitrary 3-D geometries by finite element method (FEM) simulations. An efficient raytracing algorithm was applied to the acoustic problem while the acoustic streaming interaction was modeled by a body force term in the Stokes equation. Detailed theoretical investigation of leaky surface acoustic wave-induced streaming was reported by Vanneste et al. [17].

Moreover, we demonstrated the broad applicability of SAW streaming for various biological and chemical applications. First, we developed a microfluidic device on a planar surface using the techniques mentioned above. Here, we combined the SAW technique with thin film resistance heaters for a biological analysis chip with integrated DNA amplification by polymerase chain reaction (PCR) and hybridization. The necessary volume for this was as low as 200 nL [18,19]. Moreover, within the last ten years or so, our group did not only employ surface acoustic waves for mixing or handling of small volumes but also to elucidate biophysical questions. One of the very exciting applications was the investigation of the highly non-linear shear stress dependent unfolding of the “von Willebrand factor” (VWF), a protein being omnipresent in our circulatory system and necessary to start primary hemostasis. By mimicking a wide range of blood flow conditions with direct visualization, the conformational dynamics of this protein in the presence of any adsorbing surface were shown to be of a reversible nature [20]. Along the same lines, employing the combination of a SAW based microfluidic reactor with an atomic force microscope, we studied the relaxation of stretched VWF bundles formed by hydrodynamic stress. We found that the dynamical response of the network is well characterized by stretched exponentials, from which the slowest one is dominated by the internal conformations and effective friction within the bundle. These findings on VWF-VWF-interaction under shear became possible due to broad range of tunable shear forces with such a hybrid reactor [21]. Moreover, for the interaction of VWF with melanoma cells and the matrix protein collagen type I, we also applied the open system based on hydrophilic tracks and SAW as a nanopump [22]. Within the last few years, we have developed a miniaturized (~100 μL) lab-on-a-chip hybrid system which allows for the quantification of cell adhesion under dynamic conditions which are comparable to those of physiological relevance. Amongst others, we investigated an osseointegration model with Saos-2 cells [23]. Just recently, we also demonstrated the positive effects of very low amplitude SAW on the stimulation of cell migration [24]. Our data clearly exhibits the SAW induced, dynamic mechanical and electrical stimulation and directly promotes the cell growth. Thus, we firmly believe that this SAW based cell stimulation method offers a powerful platform for future medical treatment.

The many different SAW based application examples as summarized above, are quite far and advanced from simple mixing at low Reynolds numbers. Nevertheless, this apparently simpler SAW task, which was demonstrated almost 10 years ago is still of high interest, as for example reproducible and parallelizable systems are needed for, e.g., the synthesis of nanoparticles (NP). For some particle systems SAW atomization has been demonstrated before [25].

Here, we now focus on controlled and reproducible mixing for NP production based on SAW induced chaotic advection [9,15], based on propagative waves and Rayleigh streaming with a typical decay length at the solid-fluid interface of about 10 wave lengths [16]. To combine the microfluidic NP production reactor and SAW mixing, we use a polydimethylsiloxan (PDMS) Y-shaped channel, which is mounted directly on a SAW chip. This hybrid approach gives us the possibility of inducing acoustic streaming in any desired direction with respect to the mean flow in the microfluidic channel. We are thus able to mix two fluids in a highly controllable and fast way by relying on chaotic advection. In contrast to previous works [9,26,27,28] we investigate the mixing quality as a function of tunable system parameters, such as applied SAW power, volume flux and fluid viscosity.

Finally, we demonstrate the advantages of our hybrid approach for the production of therapeutic nanoparticles. The basic mechanism for particle formation is the formation of polyplexes by mixing of cationic polymers in one solution and negatively charged nucleic acid in the second [29,30]. The microfluidic technology, due to the ability of rapid mixing of fluids on the nanoliter scale, is highly beneficial for obtaining well defined samples of organic nanoparticles. Fast mixing provides homogenous reaction environments, which in the case of macroscopic hand mixing is affected by mixing kinetics and results in spatial and temporal inhomogeneities of particle formation. Furthermore, homogenous reaction environments can lead to a more reliable formation of particles with decreased size and polydispersity compared to particles generated by conventional methods. Here, mixing is achieved by diffusion alone due to the low Reynolds number regime. In our approach, we are using SAW driven mixing to increase the speed of the mixing process by folding streamlines and thus reducing the distance for diffusion in order to decouple the process of particle formation and the mixing kinetics even further. Two different cationic systems for a potential transfection use were tested. The systems are chosen to represent different types of transfection agents (cationic polymers and lipids) and different synthesis methods (mixing of aqueous solutions and solvent exchange approach). The first system is a commonly used branched polyethylenimine (bPEI)/plasmid DNA (pDNA) particle system, where the relatively huge polymer bPEI (25 kDa) with high nitrogen content and multiple positive charges at neutral pH, forms polyplexes by ionic interaction with the negatively charged nucleic acid after mixing [31,32,33]. The second system are the stable nucleic-acid-lipid particles (SNALPs) [34,35,36]. Those small particles are dedicated for short siRNA oligonucleotide encapsulation and are synthesized by a solvent exchange method [37]. An additional polyethylene glycol shielding layer on the mono-nucleic acid/lipid particles (mNALPs) surface results in an increased particle stability under biomimetic conditions and prevents unspecific interactions with the cell membrane. Here we will focus on a particular formulation protocol leading to mNALPs that were shown to consist of a single siRNA duplex covered by a single, highly curved lipid bilayer [38]. mNALPs by design are already limited in size (~30–38 nm) but the production does not always lead to monodisperse particle distributions due to poorly controlled kinetics during manual hand mixing. The later leads to formation of clustered structures and hence more reliable mixing methods for synthesis by self-assembly are demanded.

## 2. Materials and Methods

### 2.1. SAW-Chip and Microchannel Fabrication

For the generation of the microfluidic flow, we fabricated so called “tapered” Inter-Digital Transducers (IDT) [39] of Ti-Au-Ti (5 nm-50 nm-5 nm height) on a LiNbO_3_ (128° rot Y-cut) substrate. The tapering of the IDT results in a band of excitable SAW frequencies ranging between *f* ~79.5–82.5 MHz on the same chip. Electronically, such a tapered transducer acts as a passband filter. A nice side effect for research purposes is, however, that by variation of the applied radio frequency, SAW are generated in the form of a narrow jet at different positions along the IDT aperture. Hence, this spatial variation of the sound path allows us to also control the position of the acoustically induced fluid jet. Signal generators (CellEvator, Advalytix, München, Germany) with customized Lab-View-based control software and standard SMA-connectors were used. Typical voltages are 5.6 V (peak) according to *P* = 25 dBm. We decided to design the setup in a way that can be easily reproduced and installed in multiple labs. Thus, we chose a frequency in the frequency range typically exploited for SAW-mixing [40]. Lower frequencies and thus a higher wave length could lead to undesired standing waves or reduced mixing efficiency. In contrast, to employ higher frequencies requires more sophisticated techniques, which contradicts our intention for accessibility for labs without decent ultra-high-frequency equipment. To protect the multi-finger electrodes, a SiO_2_ coating was sputter-deposited on top of the IDT structures. At the same time the LiNbO_3_ substrate acts as the bottom of our microchannel.

The Y-shaped elastomer microchannel consists of a simple polydimethylsiloxane (PDMS) single layer and is fabricated by standard soft lithography [41]. Two equally sized inlets with a cross section of 100 µm × 100 µm converge in a rectangular main channel of 200 µm width. The two solvents are injected into the channel at identical flow rates F/2. In the 3-in-1 channel, three inlets with cross sections of 50 µm × 50 µm and 20 µm × 50 µm for the outer inlets and the middle inlet, respectively, converge in a main channel with a cross section of 120 µm × 50 µm. The PDMS block was placed carefully on top of the chip with the designated IDT fitted precisely in the intended cavity. The distance between the first finger of the IDT and the channel wall was approximately 200 µm. The SAW coupled perpendicularly into the channel approximately 450 µm and 330 µm downstream from the junction for the Y-shaped channel and the 3-in-1 channel respectively. The block was pressed with about 0.5 mNm on the chip with an aluminum plate fastened to the bottom plate with four screws.

### 2.2. Particle Image Velocimetry Experiments

The generated flow field was characterized as described earlier [42]. In short, latex beads (Polystyrene, diameter 3 μm, Polybead^®^, Polysciences, Inc., Warrington, PA, USA) were added to the fluid in the channel. These particles were then used as tracers to follow the streamlines and make the fluid motion visible. For the analysis, the flow was recorded by a high speed camera (FASTCAM 1024PCI, Photron, Ottobrunn, Germany). At each position along the fluidic channel, 50 frames at a rate of 1000 fps were captured. A MATLAB (7.11.0.548 (R2010b), The MathWorks, Inc., Natick, MA, USA) script based on the open source PIVlab (version 1.35) toolkit [42,43,44] was employed to extract the three-dimensional velocity profile and will be described subsequently. The captured videos were analyzed in a PIVlab batch process in order to determine the local velocity profiles. The results at different positions were then stitched and the missing data points eventually recovered by a linear interpolation, ending up with layered *x-y* velocity profiles for the whole region of interest.

### 2.3. Mixing Experiments

**Figure 1:**
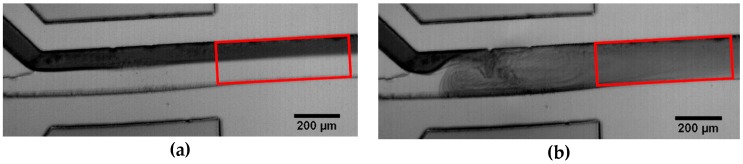
Typical micrographs of the mixing process. The Inter-Digital Transducers (IDT) is located above the channel outside of the micrograph: (**a**) Laminar flow without any applied surface acoustic waves (SAW); (**b**) Snapshot of the channel while mixing with SAW. The red box indicates the region which was analyzed with ImageJ.

To verify the SAW induced mixing quality, we used light microscopy in combination with a high speed camera (Photron). For the analysis process, one of the two fluids to be mixed was dyed with a food coloring (Patent Blue V calcium salt, 1 mM, Sigma Aldrich, St. Louis, MO, USA). To vary the fluid viscosities, different water-glycerol-mixtures were used [45]. The videos were analyzed with the public domain software package ImageJ (1.48v, National Institutes of Health, Bethesda, MD, USA) [46]. In Figure 1, we show typical micrographs illustrating the mixing process. The area highlighted by the red box indicates the analyzed region. The red box of size 200 µm × 580 µm is defined to start at the right end of the window in the PDMS channel above the IDT through all measurements. These cavities define the IDT position for all experiments. For the Y-shaped channel this results in a distance between the junction and the fluid jet of 450 µm. For the latter introduced 3-in-1 channel this distance is 330 µm. Figure 1a exhibits the typical laminar flow pattern of a microfluidic channel without any applied SAW, while Figure 1b shows a mixing pattern generated by SAW. The 8 bit gray scale videos yields discrete values ranging from 0 (black) to 255 (white). To quantify the mixing quality, we introduce a mixing parameter $\tilde{M}$, given by the mean gray scale value of the analyzed region divided by the standard deviation:

(1) $$\tilde{M}=\frac{\bar{X}}{\sigma }=\;\frac{\bar{X}}{\sqrt{\frac{1}{n-1}\sum_{i=1}^{n}{\left(X_{i}-\bar{X}\right)}^{2}}}\;$$

Here, $\bar{X}$ is the arithmetic mean grey value, σ is the standard deviation, *n* is the number of pixels and $X_{i}$ is the gray scale value at the position *i*. A homogenous distribution of the gray scale values results in small values of σ, which in turn leads to large values of σ^−1^. To compensate the influence of slightly different illumination for the different images and to ensure comparability, σ^−1^ is scaled with $\bar{X}$. To ensure comparability and intuitive understanding of the according results, we normalize the difference of the mixing parameter $\tilde{M}$ for SAW-Mixing and diffusive mixing (SAW off) to the interval [0, 1] named “mixing efficiency”:

(2) $$M=\frac{\tilde{M}-{\tilde{M}}_{\min}}{{\tilde{M}}_{\max}-{\tilde{M}}_{\min}},$$

where ${\tilde{M}}_{\min}$ is the value of the unmixed and ${\tilde{M}}_{\max}$ the value of the mixed state.

### 2.4. Nanoparticle Synthesis and Sample Evaluation

For the production of bPEI polyplexes all solutions have been degassed for 15 min at approximate 8 mbar. Purified water (evoqua water technologies) was filled into three 1 mL syringes (Norm-Ject^®^ Tuberkulin, Henke Sass Wolf, Dudley, MA, USA) equipped with Hamilton needles (ga27/90mm/pst4, Hamilton, Bonaduz, Switzerland) and placed into a syringe pump (LA-160, Landgraf Laborsysteme HLL GmbH, Langenhagen, Germany). The needles were inserted into the tubes and the whole system was washed with purified water for 15 min to stabilize the flow and to remove any unwanted substances from the channels at a flow rate of *F* = 600 µL/h. Then, the two syringes connected to the outer inlets were replaced carefully to avoid bringing gas into the system. The solutions in these syringes contained 0.12 mg/mL branched polyethylenimine (bPEI) (PEI25, Sigma Aldrich, St. Louis, MO, USA) and 0.156 mg/mL plasmid DNA (pDNA) (pCMVLuc, Plasmid Factory, Bielefeld, Germany) in purified water respectively. Using a flow rate of *F* = 150 µL/h, a reference mixed diffusively and a fraction mixed by SAW streaming was collected. Each sample was stirred for 8 min. In addition, a third sample entitled “hand mixing” was prepared manually by adding 7 µL of the above mentioned pDNA and bPEI solutions to 7 µL of purified water by vigorous pipetting. All samples have been incubated for 45 min and were measured afterwards by Dynamic Light Scattering (DLS, Zetasizer Nano ZS, Malvern Instruments, Malvern, UK) using DTS1070 folded capillaries.

The chloroform solutions of lipids used for mNALP synthesis, namely: DOTAP, 1,2-dioleoyl-3-trimethylammonium-propane (chloride salt); DOPE, 1,2-dioleoyl-sn-glycero-3-phosphoethanolamine; DOPC, 1,2-di-(9Z-octadecenoyl)-sn-glycero-3-phosphocholine; DSPE-PEG(2000), 1,2-distearoyl-sn-glycero-3-phosphoethanolamine-N-[methoxy(polyethylene glycol)-2000] (ammonium salt) and DSPE-PEG(2000)-FolA,1,2-distearoyl-sn-glycero-3-phosphoethanolamine-N-[folate(polyethylene glycol)-2000] (ammonium salt) were purchased from Avanti Polar Lipids Inc. (Alabaster, AL, USA). Since DNA is less prone to nuclease degradation, the 21 bp double stranded DNA was used as physicochemical model of siRNA. The single-stranded DNA oligonucleotides were purchased from Eurofins Genomics (Ebersberg, Germany). Oligos were lyophilised and HPLC-purified by the company.

Two 21 base complementary sequences, strand 1: 5′-CCA-ACA-GTA-AAA-GGA-ATA-TCC-3′ and strand 2: 5′-GGA-TAT-TCC-TTT-TAC-TGT-TGG-3′ were used. The 5′ end of strand 1 was conjugated with Cy3 dye to facilitate the further sample evaluation by fluorescence correlation spectroscopy (FCS, Zeiss, Jena, Germany). The oligonucleotides were annealed in equimolar solution of 20 µM in 30 mM HEPES + KOH, 100 mM KCl, 2 mM MgCl_2_, 50 mM NH_4_Ac, pH = 7.5 annealing buffer. Annealing was done by 2 min incubation at 96 °C and subsequent slow cooling at room temperature. The 21 bp dsDNA product was dialysed against 20 mM TRIS + HCl, 2mM EDTA (2X TE) pH = 8 buffer and stored at −20 °C.

The mNALP particles were formed by a ten-fold dilution of molecular solutions of all components (lipids and dsDNA; 50% (*v/v*) IPA/H_2_O) in de-ionised water, as described in detail in [24]. Firstly, the required amount of all lipids (chloroform suspensions) in the molar ratios 1:5:6:1.2 DOTAP:DOPE:DOPC:DSPE-PEG(2000) were placed in glass vials, then dried under nitrogen flow and finally placed in a vacuumized exsiccator overnight. Then, the lipids were resuspended in an isopropanol/deionised water mixture (60% (*v/v*) IPA/H_2_O) to the final DOTAP 300 µM concentration. The lipid solution was sonicated and gently mixed with 21 bp dsDNA stock solution (20 µM), IPA and H_2_O to a final 400 nM concentration of dsDNA, 84 µM of DOTAP and 50% (*v/v*) IPA/H_2_O in another glass vial. This final solution was diluted ten times in de-ionised water which finally led to the particle formation. For comparison reasons, the actual samples were prepared employing three different mixing methods: Hand mixing by dropwise dilution and vortexing; and SAW mixing on a microfluidic chip (3-in-1-geometry); and diffusive mixing in the channel of the same geometry. The flow rates were controlled with TSE systems syringe pumps (model 540200, TSE Systems, Bad Homburg vor der Höhe, Germany) and kept at *F* = 0.05 mL/h and *F* = 0.45 mL/h for the molecular solution and water, respectively. For the acoustic mixing case, the SAW was generated at an IDT frequency of *f*_SAW_ = 81.2 MHz and a constant power of *P*_SAW_ = 27 dBm. All solutions and liquids used for microfluidic synthesis were degased in a vacuumized exsiccator.

Fluorescence Correlation Spectroscopy (FCS) was used to evaluate the quality, the size and the encapsulation efficiency of the lipid-DNA complexes. For the FCS experiments, a Zeiss LSM510 ConfoCor2 confocal microscope (Zeiss, Jena, Germany) with a C-Apochromat 40×/1.2 NA water immersion objective was used. The measurements were performed using a λ = 543 nm HeNe laser excitation and a bp 560–615 nm emission filter on Cy3-labelled samples. 21 bp DNA duplex was taken as a size reference. For both mNALP samples, the residual, freely diffusing dsDNA was seen in the system. Due to this, the model with the two 3D diffusion components (one for mNALP one for free dsDNA) and one triplet component was used. The model is described by following equation:

(3) $$G\left(\tau \right)=\;A_{0}+\frac{1}{n{\left(\tilde{F}+\alpha \left(1-\tilde{F}\right)\right)}^{2}}\left(1+\frac{Te^{-\tau /\tau_{\mathrm{trip}}}}{1-T}\right)\left[\frac{1}{\left(1+\tau /\tau_{1}\right)}\frac{1}{\sqrt{1+\tau /\left({\mathrm{SP}}^{2}\tau_{1}\right)}}+\alpha^{2}\frac{1-\tilde{F}}{\left(1+\tau /\tau_{2}\right)}\frac{1}{\sqrt{1+\tau /\left({\mathrm{SP}}^{2}\tau_{2}\right)}}\right]$$

Here, *A*_0_ denotes an offset, *n* the effective number of particles in the confocal volume (*n* = *n*_1_ + *n*_2_), τ_1_ the diffusion time (correlation time) of particle species 1; τ_2_ the diffusion time (correlation time) of particle species 2. $\tilde{F}$ represents the fraction of molecules of species 1 ($\tilde{F}\;$= *n*_1_ /(*n*_1_ + *n*_2_), α the relative molecular brightness of particles 1 and 2 (α = *q*_2_/*q*_1_). SP is a structural parameter, *T* the fraction of particles in triplet state and τ_trip_ the characteristic residence time in the triplet state. The particle fraction $\tilde{F}$, as being obtained directly from the fit was taken as a measure of the encapsulation efficiency.

## 3. Results and Discussions

The structure of this section is as follows: First, we characterize the mixing process of aqueous solutions using a Y-shaped channel in general and estimate the mixing time. Second, we elucidate the role of flow rate and applied SAW power and their importance and influence for an optimal mixing effect. Next, we study the role of viscosity: Based on the findings of the role of flow velocities, we therefore measure the acoustic streaming velocity as a function of the viscosity. For the production of particles with a lipid shell and a DNA core, often mixing of different solvents is necessary. Thus, in the following step, we characterize the SAW induced mixing of both isopropanol and ethanol with water. Finally, we demonstrate the potential of the SAW mixing technique for the production of therapeutic nanoparticles using established systems. Here, we discuss hurdles like unintended complex formation at the interface of the two liquids and how to overcome those by using an additional separation layer in a 3-in-1 channel with three inlets. Finally, we apply the SAW-mixing to the fabrication of nanosized polyplexes composed of bPEI and pDNA, as well as to the mNALP system.

### 3.1. Mixing Process

**Figure 2:**
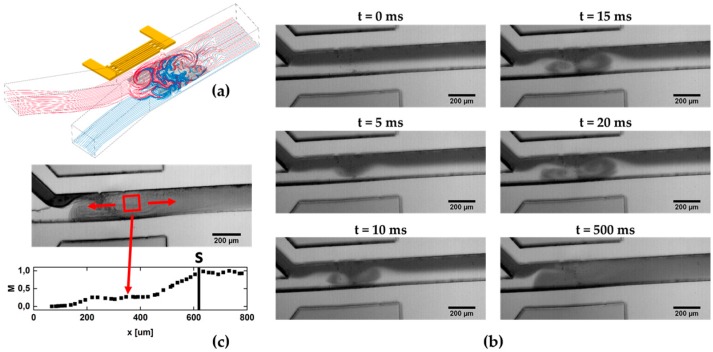
(**a**) Sketch of the principle setup: due to acoustic streaming two solvents are mixed by chaotic advection; (**b**) Time sequence of the mixing process after launching of the SAW; (**c**) Using the measure *M* for the mixing efficiency, the distance where the fluids are completely mixed are determined in order to estimate an upper limit of the mixing time.

In Figure 2a, we sketch the mixing process in a Y-shaped channel employing acoustic streaming. As described earlier [15,16], the mixing is based on chaotic advection, by generating a fluid jet perpendicular to the mean flow direction. Under the influence of the SAW induced acoustic streaming, the streamlines become folded which in turn contributes to an increase of the diffusive mixing efficacy. In Figure 2b, a time sequence of the mixing process after switching on the SAW is shown. At *t* = 0, the SAW is not yet coupled into the fluid and the picture is the same as for laminar flow of the two fluids from left to right. In this case, only diffusive mixing takes place at the fluid-fluid interface. At *t* = 5 ms, the SAW already generates acoustic streaming in the fluid as can be seen by the colored fluid being pushed into the transparent water. During the ongoing process, two vortexes are formed with opposite sense of rotation. The last micrograph, finally shows the stationary state, where the two solutions are completely mixed.

Due to the complexity of the streamline geometries and the two vortexes of opposite sense of rotation, it is difficult to define a specific value for the mixing time of the fluids. Hence, we conservatively estimate an upper limit for this mixing time, by determining the time until the two volume elements of the fluids are completely mixed under stationary conditions. To do so, we measure the distance that the volume elements travel before they are completely mixed and divide this distance by the mean flow velocity. Figure 2c shows the mixing parameter *M*, determined from the region indicated by the red box as function of the position along the channel until it reaches a constant value at a certain position *s*. Given the mean flow velocity *v* in the channel, the mixing time *t*_M_ can now be estimated:

(4) $$t_{M}=\;\frac{s}{v}$$

For our chip geometry, the result is a mixing time *t*_M_ = 189 ms for two aqueous solutions at a flow rate of 0.2 mL/h. As mentioned, this time depends on the flow rate, as a conservative upper limit and can still be reduced, as will be shown in Section 3.4 and Section 3.5.

### 3.2. Role of the Flow Rate and RF-Power

**Figure 3:**
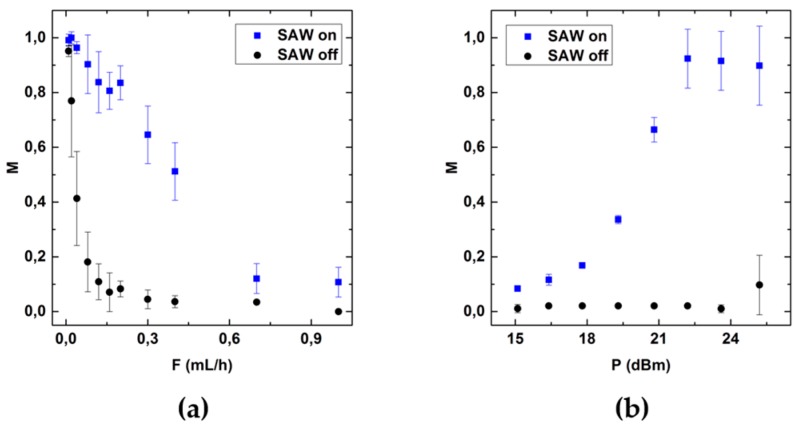
(**a**) Mixing quality as a function of the flowrate *F*; (**b**) Mixing quality as a function of SAW power *P*. For *P* > 21 dBm sufficient mixing is achieved. The data point and error bars represent the mean and the standard deviation respectively of at least three experiments.

**Figure A1:**
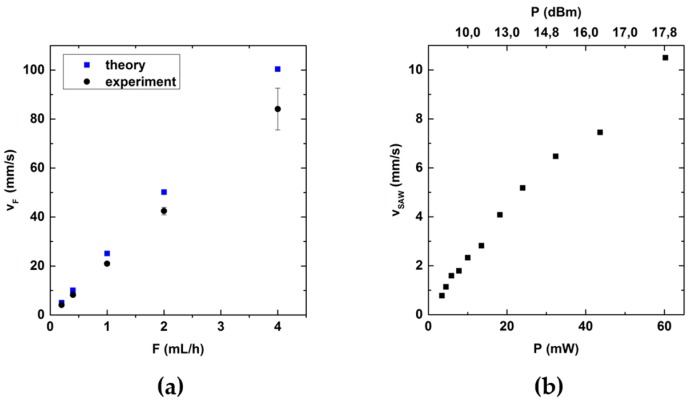
(**a**) Maximal flow velocity in the center of the channel as being measured and calculated; (**b**) Induced SAW-streaming velocity in a bulk volume (compare Figure 4a) parallel to the chip surface as a function of SAW power.

The two most important system parameters having a large influence on the mixing quality *M*, are the flow rate *F* and the RF-power *P*. We measured the maximal flow velocity in the center of the channel as a function of flow rate *F* (see Figure A1a). The blue squares are taken from particle image velocimetry (PIV) measurements, the black dots are the results of theoretical calculations [47]. As expected, we clearly see a linear dependence of the flow velocity on the flow rate in our experiments as well. The systematically smaller values for the experimental data are due to the fact that in the model, only the exact center of the channel is considered, whereas experimentally, the evaluation of the velocity also includes regions slightly away from the center and hence regions of slightly smaller velocities. Figure 3a shows *M* as a function of the flow rate at a constant power of *P* = 25 dBm. In the regime 0.01 mL/h < *F* < 0.6 mL/h, *M* decreases linearly, whereas for higher flow rates *F* > 0.6mL/h, almost no mixing is further observed.

Moreover, Figure 3b shows *M* as a function of the RF-power *P* at the constant flow rate of *F* = 0.2 mL/h. For *P* < 19 dBm, almost no mixing is observed. For 18 dBm < *P* < 22 dBm, the mixing quality *M* increases and eventually saturates for higher values *P* > 22 dBm, indicating that the fluids are completely mixed. To understand this, we recall the dependency of acoustic streaming velocity and SAW power *P*. Figure A1b shows the induced SAW-streaming velocity *v*_SAW_ in bulk parallel to the chip surface as function of *P*. In accordance to previous work [16] *v*_SAW_ increases linearly with increasing *P*.

The data in Figure 3 allows us to explain the upper limit for the flow rate where suffiecient mixing is achieved: a constant SAW-power, e.g., *P* = 25 dBm, results in a constant velocity *v*_SAW_, e.g., *v*_SAW_ = 7 mm/s, as determined by PIV measurements in the channel. As it turns out, optimum mixing occurs if the main flow velocity *v_F_* and *v*_SAW_ are equal. A further increase of *v*_SAW_ does of course not yield better than optimum results. For $r≔\frac{v_{\mathrm{SAW}}}{v_{F}}\;$<< 1, no sufficient mixing is achieved.

### 3.3. Role of Viscosity

**Figure 4:**
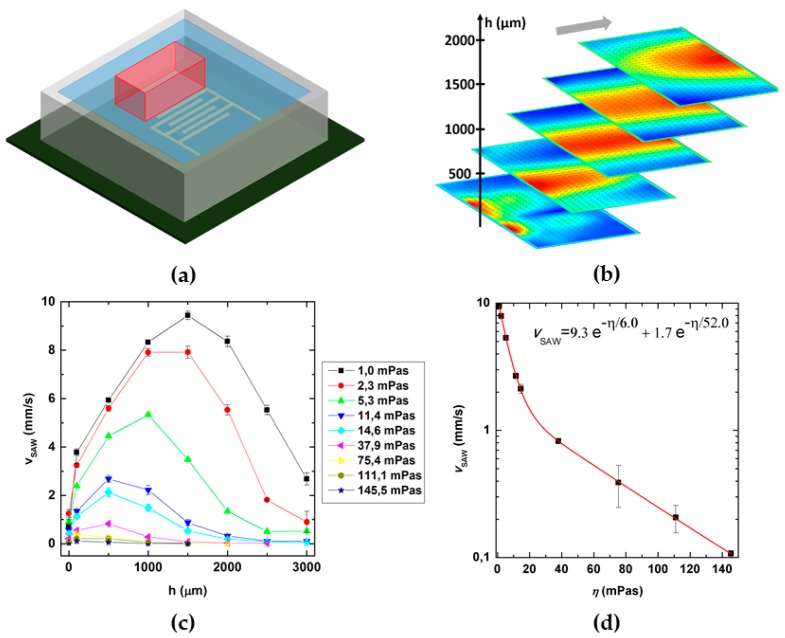
(**a**) Sketch of the used setup. A PDMS wall (grey) is placed on the SAW-chip and filled with a fluid. The red area shows the measured region in front of the aperture starting at the first finger of the IDT; (**b**) Velocity field for different heights, measured by particle image velocimetry (PIV). The position of the jet changes with height. The color scale gives the distribution of the velocity in each layer (blue = minimal, red = maximal). The general in plane flow is from left to right as indicated by the grey arrow; (**c**) Mean SAW velocity from (**b**) dependent on the height in the fluid for different viscosities. The maximal velocity decreases with increasing viscosity. The overall shape of the graph is caused by a viscosity dependent Rayleigh angle and fixed positions of the analyzed region; (**d**) The maximal SAW velocity declines bi-exponentially with increasing viscosity of the fluid. The data point and error bars represent the mean and the standard deviation respectively, of at least three experiments.

**Figure A2:**
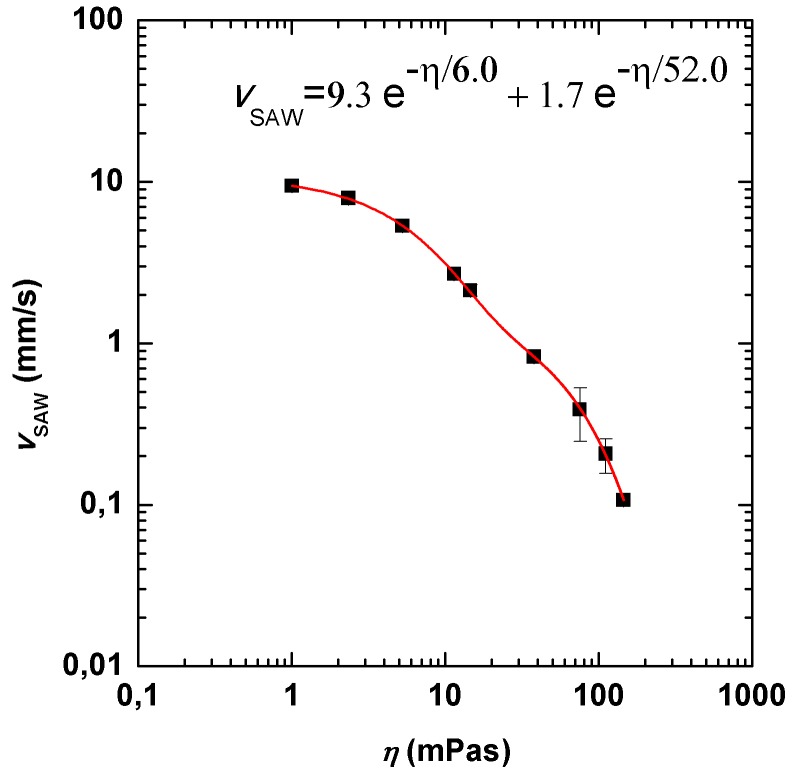
Maximal flow velocity as function of viscosity (data of Figure 4d) in a double logarithmic scale.

Here, we elucidate the role of the fluid viscosity on the SAW induced mixing process. To do so, we measure both the SAW velocity *v*_SAW_ as well as the mixing quality *M* as function of viscosity η. For the SAW velocity experiments, a PDMS wall was placed on a SAW-chip and filled with water-glycerol mixtures of different dilution and thus different viscosities as illustrated in Figure 4a. The red cuboid next to the IDT marks the volume, which is evaluated by PIV measurements. Typical flow fields obtained from such measurements are shown in Figure 4b. Obviously, the lateral velocity field in the fluid is a function of the height above the chip. Along the channel, the position of the region of highest velocities shifts to positions further “downstream” with increasing height. This is a consequence of the existence of the Rayleigh diffraction angle, when the SAW couples into the medium. For LiNbO_3_ and water, this angle between the jet and the vertical is approximately 22°. From the measurements, we determine the magnitude of the speed as a function of height as being depicted in Figure 4. Here, we show the extracted maximum speed of the fluid as a function of the position along the channel for various fluid viscosities η. With increasing viscosity, the maximum speed decreases. For a viscosity of η = 1.0 mPas, which corresponds to water at 20 °C, the fluid velocity reaches almost *v*_fl_ = 10 mm/s. For viscosities higher than η = 25 mPas, the velocity drops below *v*_fl_ = 1 mm/s. The maximal velocity as function of viscosity is described best by a bi-exponential decay, as can be seen in Figure 4d. Theoretically an exponential decay with only one decay constant is expected [48]. We furthermore compare our data with a very recent publication on simulations of acoustic streaming in droplets [49]. If plotted in a double logarithmic scale, our data for *v*_SAW_ (η) match the values in this publication extremely well (compare Figure A2), though Riaud et al. claim a power law to fit the data best. Alternatively, the deviations from an exponential decay here could be attributed to be caused by a second force which becomes dominant for low viscosities and thus low friction. This contribution strongly depends on the boundary conditions like microchannel dimensions and materials and has to be determined for each designated setup separately. An increased temperature due to increased dissipation may lead to a decreased viscosity, which could explain the appearance of a second decay constant. However, in the most relevant viscosity range for SAW mixing of solutions in microchannels (η = 40 mPas) *v*_SAW_ is sufficiently described by a single exponential decay.

**Figure 5:**
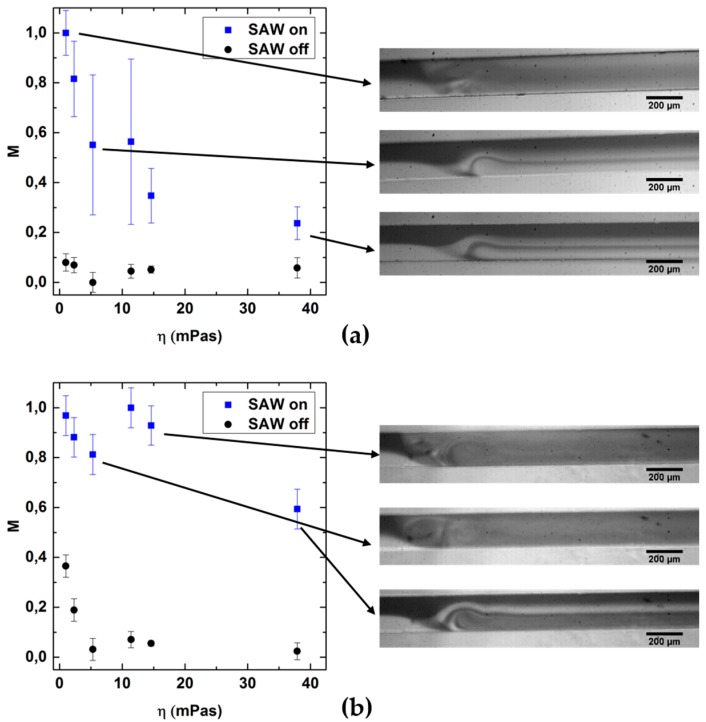
Mixing quality as a function of the viscosity for a constant flow rate of (**a**) *F* = 0.2 mL/h; and (**b**) *F* = 0.02 mL/h. Insets: Typical micrographs of the mixing area. The data point and error bars represent the mean and the standard deviation respectively, of at least three experiments.

To investigate the mixing of solvents with higher viscosities, we used the same setup, the same parameters and the same procedures as described above and characterized the mixing quality *M* as function of η. Figure 5 shows these mixing results for water-glycerol-mixtures of different viscosities. First, in Figure 5a, we show *M* as a function of the viscosity η for a constant flow rate of *F* = 0.2 mL/h and *P* = 25 dBm in a Y-shaped channel of the same geometry. *M* decreases rapidly with increasing η. For η > 5 mPas, no sufficient mixing can be detected. This can be understood by a reduced SAW-velocity (see Figure 4d) in combination with a reduced diffusion coefficient, being indirectly proportional to η. Typical micrographs of the according situations point up this behavior. For η = 1 mPas, a good mixing quality can be achieved and the distribution is homogeneous. For higher η, the distribution of the dye is not homogenous but rather, exhibits a striped pattern. Here, due to the low *v*_SAW_ and the large attenuation, the fluid jet only “squeezes” and thus folds the streamlines, as can be seen in the insets in Figure 5a.

Consequently, for η > 5 mPas and to create a sufficiently low ratio *r*, the main flow velocity *v_F_* has to be significantly reduced. In Figure 5b proof is shown that in fact, good values of *M* are achieved by reducing the main flow by a factor of ten to *F* = 0.02 mL/h.

### 3.4. Mixing Aqueous Solutions with Ethanol and Isopropanol

**Figure 6:**
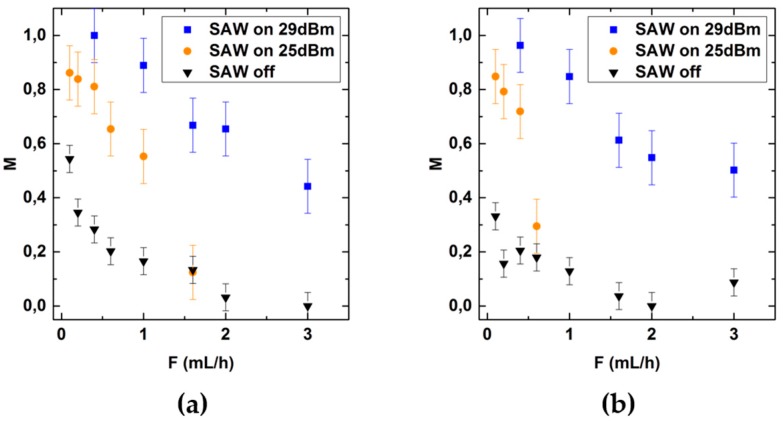
Mixing quality as a function of flow rate *F* for: (**a**) Ethanol; and (**b**) Isopropanol respectively, with water for *P* = 25 dBm and *P* = 29 dBm. The data point and error bars represent the mean and the standard deviation respectively of at least three experiments.

The fabrication of therapeutic nanoparticles (TNP) with a lipid shell and nucleic acid core in aqueous solutions requires effective mixing of nonpolar solvents with aqueous solutions. To characterize the mixing efficiency as function of *F* and *P* for such systems, we here characterize the mixing of water with ethanol (1.2 mPas at 20 °C) and isopropanol (2.4 mPas at 20 °C) respectively, in the same Y-channel setup as described above. Figure 6 shows the mixing quality *M* as function of the flow rate *F* for applied SAW power *P* = 25 dBm and *P* = 29 dBm.

Applying a power *P* = 25 dBm allows sufficient mixing of aqueous solutions for flow rates up to 0.3 mL/h. While this upper flow rate is comparable to the one for mixing of isopropanol and water, it is shifted to about 0.6 mL/h for ethanol and water. In contrast to the mixing of aqueous solutions, here, increasing the SAW power to *P* = 29 dBm allows for sufficient mixing at flow rates up to 1.4 mL/h. For ethanol-water-mixtures the better mixing efficiency is in accordance with the finding of Orsi et al. [30]. They attribute this effect to the increased residence time of the fluid occupying the interfacial region in the water-ethanol case, due to an increased viscosity of the mixture at the interface compared to the pure solvents. The difference in mixing quality for water-isopropanol mixtures compared to water-ethanol mixtures for *P* = 25 dBm is in accordance with a higher viscosity of isopropanol compared to ethanol. Moreover, the lower surface tension of ethanol and isopropanol compared to water may contribute significantly to the mixing [31]. However, we did not study the role of surface tension systematically here, as the knowledge about the applicable flow rate range seems absolutely sufficient to us from a pragmatic point of view.

### 3.5. Formation and Characterization of Therapeutic Nanoparticles

**Figure 7:**
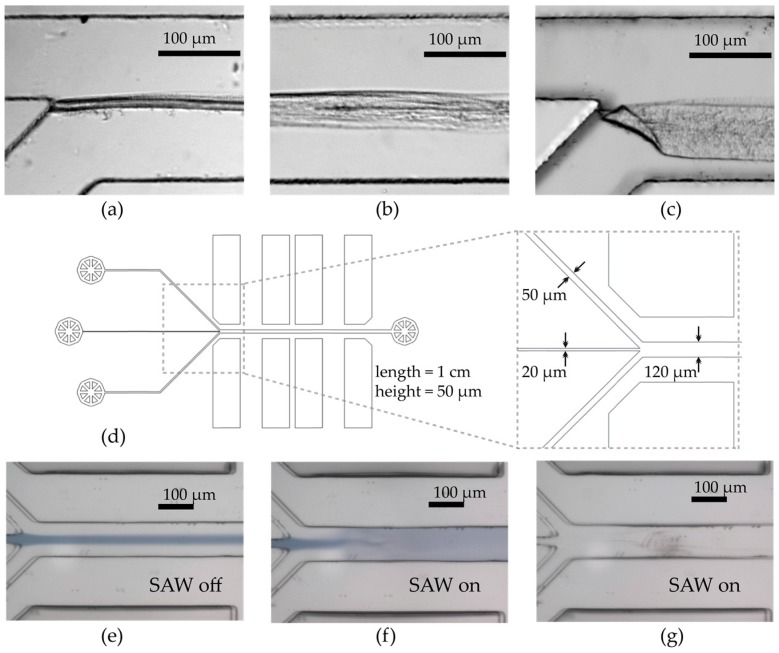
(**a**) Diffusive mixing of bPEI (upper inlet) and pDNA (lower inlet) in a Y-shaped microchannel leads to complex formation at the interface of the two fluids; (**b**) Broad layer about 10 mm downstream; (**c**) Complex layer after 24 h of incubation, i.e., without flow; (**d**) The design of the Y-shaped channel is modified to a 3-in-1 channel; (**e**) Using the 3-in-1 channel with a “protective” buffer layer (here blue) ensures the prevention of complex formation; (**f**) 3-in-1 channel with applied SAW; (**g**) Mixing of bPEI and pDNA in a 3-in-1 channel: no unwanted complex formation occurs, only some minor precipitation at the channel bottom appears without disturbing the particle formation.

In this last section, we demonstrate the applicability and the potential of SAW-mixing for the production of therapeutic nanoparticles. First, we concentrate on an unwanted and unintended complex formation at the interface of the two fluids that turned out to be very counterproductive for the NP production process. Secondly, we apply the optimized acoustic mixing technique to a commonly used polyplex system of bPEI and pDNA. Finally, we investigate the application of SAW mixing to a more sophisticated system of lipid-based mono-nucleic acid lipid particles (mNALPs) yielding very good results. To benchmark the results we here compare mixing by hand following a strict protocol, diffusive mixing using a channel as shown in Figure 7d and SAW-mixing in the same channel.

In a first step, to investigate the formation of polyplexes from cationic polymers and plasmid DNA, we mix 80 µg/mL bPEI with 100 µg/mL pDNA in the above characterized Y-shaped channel: The flow rate was set to *F* = 0.2 mL/h. Since no SAW is applied, only diffusion is thus acting on the fluids. As can be seen in Figure 7, at the interface of the two solutions close to the junction of the inlets, a thin solid structure is formed within about 150 s. The thickness of this layer increases with increasing distance downstream from the inlets. Figure 7c shows the same channel after 24 h of incubation at rest, i.e., without flow. Obviously, a thin wall had been formed by a complex formation due to the rapid and firm ionic interaction of charged bPEI and pDNA at the fluid–fluid interface. This layer of course deteriorates the ability to actively mix, employing a SAW. To minimize the area where this unintended complex formation appears, the relative distance of the IDT to the inlet in principle could be reduced. However, if the IDT is too close to the junction, the pressure increases in the inlets and the according changes of the flow profile lead again to suboptimal mixing. A more elegant and controllable resolution, is to use a 3-in-1 channel with a third inlet. The latter one is used to create a thin separation streamline of pure solvent (see Figure 7e). Thus, the first contact between cationic polymers and anionic pDNA occurs at the position where the fluid jet breaks the separation layer (see Figure 7f).

**Figure 8:**
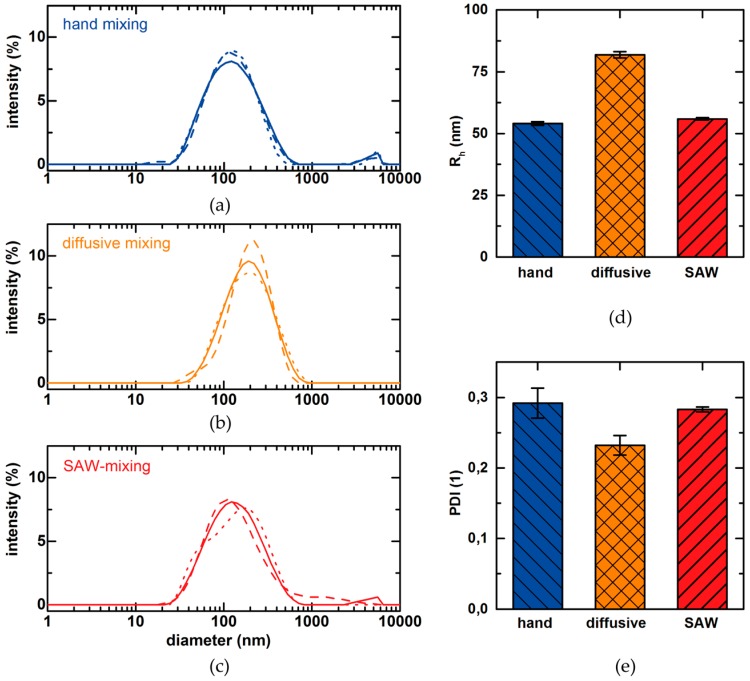
Size distribution of particles from bPEI and pDNA mixed: (**a**) in bulk; (**b**) in a 3-in-1-channel by diffusive mixing without applied SAW; (**c**) in a 3-in-1-channel with applied SAW (solid, dashed and dotted lines show the results of three measurements); (**d**) Hydrodynamic Radii *R*_h_ determined by DLS; (**e**) PDI of the according particles from (**a**–**c**).

Using such a 3-in-1 channel, we mix bPEI and pDNA at a total flow rate of *F* = 150 µL/h and an applied power of *P* = 27 dBm. As reference measurements, we fabricate particles by hand mixing as described in the materials and methods section and by diffusive mixing without applied SAW. Figure 8 shows the according size distributions as measured by DLS, the mean hydrodynamic radius *R*_h_ and the polydispersity index PDI. Hand mixing results in particle radii of *R*_h_ = 54.1 ± 0.84 nm with a PDI = 0.292 ± 0.026. Microfluidic mixing increases reproducibility in terms of the decreased PDI = 0.232 ± 0.017 and its standard deviation and results in particles with a radius of *R*_h_ = 81.8 ± 1.5 nm. Here, we like to point out the difference between the Y-shaped channel and the 3-in-1 channel: While the Y-shaped channel does not lead to the desired TNP formation by diffusive mixing, interestingly the use of the 3-in-1 channel does. This may be a consequence of the low concentrations and less steep concentration gradients within the separation layer compared to the Y-shaped channel. Finally, SAW-mixing combines the advantages of both other methods resulting in automated particle fabrication with higher reproducibility compared to hand mixing; and smaller particles compared to microfluidic mixing with radii of *R*_h_ = 55.4 ± 0.65 nm with a PDI = 0.283 ± 0.004. However, hand mixing strongly depends on handling protocols and can vary from person-to-person or lab-to-lab. Thus, SAW-mixing definitely bears the potential to improve reproducibility. Finally, we like to mention another hurdle that could appear: using SAW-mixing for very high values of power *P* can result in heating of the chip [24]. We did not optimize the setup concerning this but a cooling circuit integrated in the PDMS could easily allay heating effects here.

**Figure 9:**
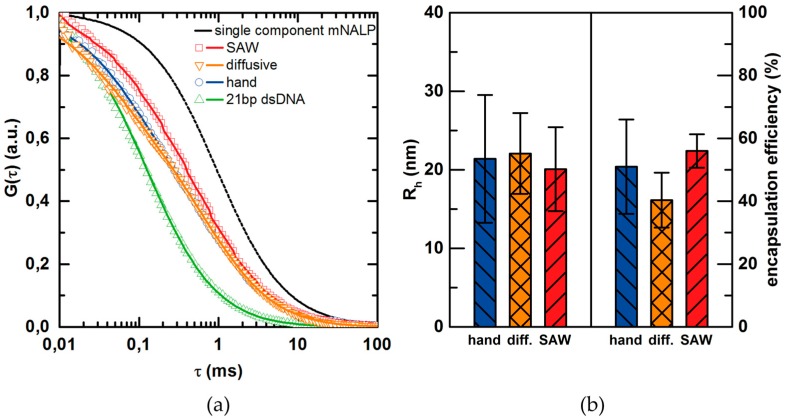
(**a**) Normalized FCS correlation curves of 21 bp dsDNA (△, green), mNALP particles prepared with hand mixing (○, blue), diffusive microfluidic mixing (▽, orange) and SAW microfluidic mixing (□, red). The solid lines show the fitted correlation curves (─). The dashed line shows (--) the correlation curve that was generated for the single component mNALP system (particles with hydrodynamic radius *R*_h_ = 19.2 nm) corresponding to 100% of DNA encapsulation. The shift towards higher diffusion times for SAW mixing relates to higher encapsulation efficiency; (**b**) The particle hydrodynamic radius and encapsulation efficiency of particles prepared by hand (blue), diffusive (orange) and SAW mixing (red) as determined by FCS.

As microfluidic polyplex formation using SAW-mixing has been demonstrated for the classic bPEI/pDNA composition, feasibility was also tested with a different more sophisticated type of TNP. Lipid-based mNALP [38] particles are synthesized in a solvent exchange method by 10-fold dilution of 50% (*v/v*) Isopropanol/water solution of lipid/nucleic acids in water. Here the interactions between molecular components, namely lipids and nucleic acids, occur when triggered by the change in the solvent quality. When the non-polar solvent (e.g., alcohol) is replaced with the polar water the hydrophobic interactions and electrostatic interactions leads to particle formation in the process of self-assembly. The kinetics of solvent exchange, thus the kinetics of mixing, is particularly crucial, as the slow alcohol/water mixing rates may interfere with particle formation and lead to formation of undesirable structures. For the purpose of our study, the same setup (3-in-1 channel) and mixing parameters as above were employed. To evaluate the quality of SAW assisted mNALP synthesis, comparative studies of both “bulk” and SAW assisted mNALP samples are performed. We use the FCS technique to investigate the basic colloidal properties, like the hydrodynamic radius *R*_h_ of the formed particles for both samples. Moreover, FCS allows us to measure the encapsulation efficiency as well. To monitor the particle formation, Cy3-labelled 21 bp dsDNA was used as physicochemical model of siRNA. It has a significant practical advantage, as DNA is less prone for nuclease degradation when compared with less stable siRNA. The naked, non-complexed dsDNA is taken as reference for monitoring the relative changes in diffusive behavior of particles. All mNALP samples are showing a complicated shape of autocorrelation curves reflecting the multiple fluorescent particle species present in the sample. All measurements which were significantly influenced by the occurrence of bright clusters were discarded from the calculations of averages. By applying the correlation model function consisting of two 3D diffusion components and one non-diffusive triplet component, we obtained the best results. One of the fitted components corresponds with the uncoated dsDNA as the diffusion time (thus the particle sizes, *R*_h_) correlates with the values obtained for reference dsDNA measurement. The second component corresponds to 3D diffusion of the particles with hydrodynamic radius *R*_h_ ~ 19 nm. It correlates well with the expected sizes of mNALP. Additionally, no significant changes in concentration of diffusing particles and counts per particle were seen. It leads to the conclusion that every diffusing particle contains a single dsDNA particle as expected for the mNALP system. For this particular binary system, the fraction of particles $\tilde{F}$ derived directly from fitting the model function to the experimental correlation function corresponds directly to the encapsulation efficiency of dsDNA in the mNALPs. The results of FCS analysis are shown in Figure 9. The normalized autocorrelation curves for samples prepared in all mixing modes and as a reference naked dsDNA sample (which corresponds to 0% encapsulation efficiency) are shown in Figure 9a. An additional theoretical correlation curve generated for the single component mNALP system (particles of hydrodynamic radius *R*_h_ = 19.2 nm; 100% encapsulation efficiency) is shown as a guide. A noticeable shift in autocorrelation towards higher diffusion times for SAW mixed samples compared to hand and diffusive mixing can be seen. This corresponds to an increase in encapsulation efficiency, as the sizes of particles are comparable for both mNALP samples (Figure 9). Moreover, the narrowing in size and encapsulation efficiency variances reflected in decreased standard deviations by a factor of about 2 (error bars in Figure 9), shows that the samples prepared by microfluidic SAW mixing are more reproducible in terms of those two parameters. The statistical significance of the results from Figure 9b was tested—the standard Student’s *t*-test was used [50]. The *t*-values are given in Table 1. We expect this benefit to increase further for more sensitive multicomponent TNP systems, as we have already reached higher reproducibility and encapsulation, even without further optimizations e.g., in terms of concentrations, concentration ratios or acoustic wave length. A direct comparison of the approach presented here: based on chaotic advection with more conventional ones reported earlier [51], based on sonication or turbulent microfluidic mixing would be highly interesting [40]. Such a systematic study could elucidate the question which mixing approach (laminar, chaotic advection or turbulent) is favorable for which particle formation mechanism.

## 4. Conclusions

We demonstrated the applicability and benefits of SAW-assisted fabrication of TNP. We elucidated the role of controllable parameters for the mixing process of aqueous solutions as well as other solvents like ethanol and isopropanol. We found the ratio of main flow velocity along the channels axis and the SAW-induced streaming velocity perpendicular to it, to be of highest importance. Consequently, for higher solvent viscosities compared to water, this ratio should be set to 1, either by increasing SAW-power or by decreasing the flow rate. Mixing of aqueous solutions with ethanol or isopropanol even allows for higher flow rates and thus higher throughput. For the formation of polyplexes from bPEI and pDNA, we introduced a separation layer to avoid an undesired complex formation prior to mixing. In contrast to Y-shaped channels, using such a 3-in-1 channel, even without SAW-mixing, allows the production of TNP by diffusive mixing. However, this leads to bigger particles compared to hand mixing, while SAW-assisted particle formation results in particles of the desired size comparable to hand mixing. Moreover, the technique enhances a more sophisticated formation of mNALPs regarding increased encapsulation as well as increased reproducibility. Summing up, we here presented a unique, promising and precisely controllable technique of SAW-assisted fabrication of therapeutic nanoparticles that opens up new possibilities for the nanomedicine community, especially for particle systems that are very sensitive to preparation conditions.

## Figures and Tables

**Table 1:** Student’s *t*-test results for data in Figure 9 (critical value of Student’s *t*-test for significance level *α* = 0.05 and *ν* = 10 degrees of freedom; *t_0.95, 10_* = 1.812. If the test value exceed the critical value the difference in experimental values are statistically significant).

Student’s *t*-Test Results	*R*_h_	Encapsulation	*R*_h_	Encapsulation
Comparison	SAW vs. hand mixing	SAW vs. hand mixing	SAW vs. diffusive mixing	SAW vs. diffusive mixing
*t*-value	1.263	4.483	1.922	14.101
